# Supplementary material for: The Skin Microbiome and Influencing Elements in Cutaneous T-Cell Lymphomas
Source: Cancers (Basel). 2022 Mar 4;14(5):1324. doi: 10.3390/cancers14051324 (PMC8909499; doi:10.3390/cancers14051324)
Supplement: Supplementary file 1 [file cancers-14-01324-s001.zip › cancers-1583886-supplementary.pdf]

Table S1. Terms used in the PubMed Search.

**PubMed Search.**	**- microbiome cutaneous lymphoma**<br>**- microbiome ctcl**<br>**- microbiome t cell lymphoma**<br>**- microbiome t cell lymphoma skin**<br>**- microbiome mycosis fungoides**<br>**- microbiome sézary syndrome**<br>**- microbio* mycosis fungoides**<br>**- microbio* sézary syndrome**<br>**- microbio* ctcl**<br>**- microbio* t cell lymphoma skin**<br>**- microbio* cutaneous lymphoma**<br>**- antimicrobial peptides CTCL**<br>**- antimicrobial peptides cutaneous t cell lymphoma**<br>**- antimicrobial peptides mycosis fungoides**<br>**- antimicrobial peptides sézary syndrome**<br>**- staphylococcus aureus cutaneous t cell lymphoma**<br>**- staphylococcus epidermidis cutaneous t cell lymphoma**<br>**- staphylococcus epidermidis mycosis fungoides**<br>**- antibiotic cutaneous t cell lymphoma**<br>**- filaggrin t cell lymphoma**<br>**- filaggrin mycosis fungoides**
